# Supplementary material for: Efficacy of a New Low-Protein Multimedia Diet App for PKU
Source: Nutrients. 2022 May 24;14(11):2182. doi: 10.3390/nu14112182 (PMC9182776; doi:10.3390/nu14112182)
Supplement: Supplementary file 1 [file nutrients-14-02182-s001.zip › nutrients-1731954-supplementary.pdf]

LOW PROTEIN LABEL READING

For the following questions, circle the answer you think best fits that product.
Please answer all questions.

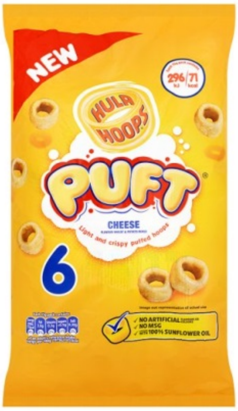

This product contains protein 1.3g/packet.

How many exchanges would you count for this product?

a. Not allowed
b. 1 exchange
c. 1½ exchanges
d. 2 exchanges
e. Exchange-free so I don't need to count it
f. Other amount (write here) __________________
g. Unsure

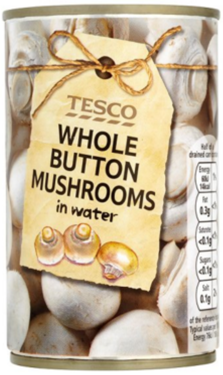

These mushrooms contain protein 2.1g/100g.

How many exchanges would you count for this product?

a. Not allowed
b. 2 exchanges
c. 2½ exchanges
d. Exchange-free because mushrooms are exchange-free.
e. Other amount (write here) __________________
f. Unsure

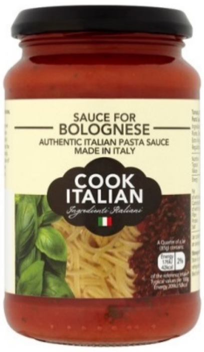

Ingredients:
Italian tomatoes (76%), tomato purée, onion (3%), sugar, carrot (2%), water, extra virgin olive oil, Basil, Salt, acidity regulator: lactic acid.

This jar of sauce contains protein 1.2g/100g.

How many exchanges would you count for this product?

a. Not allowed
b. 1 exchange
c. 1½ exchanges
d. 2 exchanges
e. Exchange-free because all the ingredients are exchange-free.
f. Other amount (write here) __________________
g. Unsure

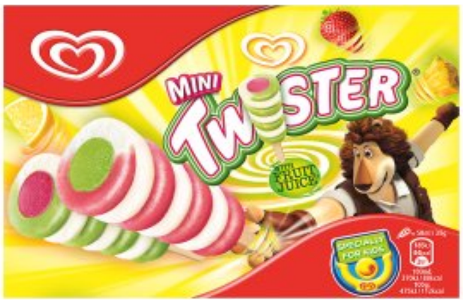

	Typical analysis/100g	Typical analysis/ 39g lolly
Protein	0.6g	<0.5g

How many exchanges would you count for 1 ice lolly?

a. Not allowed
b. ½ exchange
c. 1 exchange
d. Exchange-free if eat one only per day;
   ½ exchange if eat 2 per day.
e. Exchange-free
f. Other amount (write here) _________________
g. Unsure

	Per oatcake	Per 100g
Protein	1.0g	11.2g

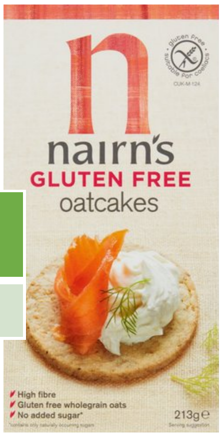

How many exchanges would you count for 1 oatcake?

a. Not allowed
b. ½ exchange
c. 1 exchange
d. 1½ exchanges
e. Exchange-free
f. Other amount (write here) ______________
g. Unsure

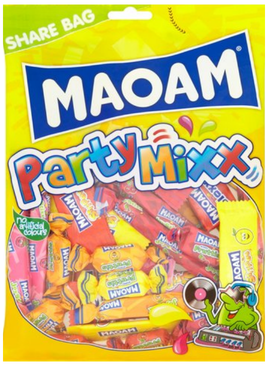

	Per 100g	Per portion (25g)
Protein	1.0g	<0.5g

Can you to eat these sweets as an exchange-free food?

a. Yes Why? ______________________________
b. No Why not? _____________________________
c. Unsure

Ingredients:
Sugar, glucose syrup, palm fat, humectant: sorbitol syrup, acid: citric acid, gelatine, flavouring.

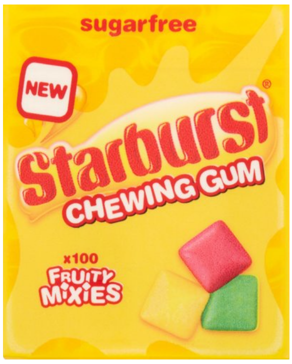

Ingredients:
Sweeteners: xylitol, sorbitol, mannitol, aspartame, acesulfame K, gum base, thickener: gum arabic, flavourings, humectant: glycerol, emulsifier: soybean lecithin, acids: citric acid, malic acid, colour E172, glazing agent carnuba wax, antioxidant BHA, colours E100, E133.

The label says the protein content of these sweets is 0g/100g.

Are these okay for you to eat?

a. Yes Why? ______________________________
b. No Why not? ______________________________
c. Unsure

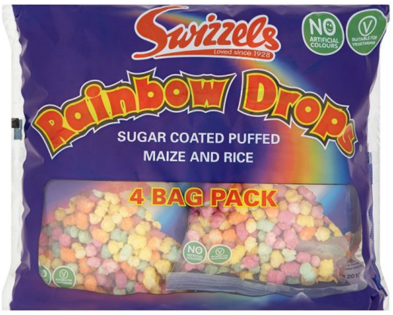

Ingredients:
Sugar, maize, rice, glucose syrup, flavourings, colours: beet red, copper chlorophyllin, lutein, paprika.

There was no protein content on the label of this product. Could you eat this?

a. Yes it's okay
b. Not allowed.
c. I would ask my dietitian
d. Could eat a small amount
e. I would count it as an exchange
f. Other: (write here)__________________________
g. Unsure

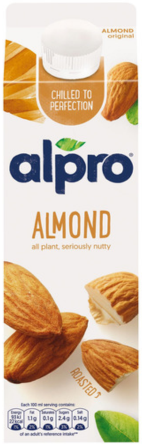

	**Per 100ml**
Protein	0.4g

If you had 200ml of this product how many exchanges would you count for this?

a. Not allowed
b. ½ exchange
c. 1 exchange
d. Exchange-free
e. Other amount (write here) __________________
f. Unsure

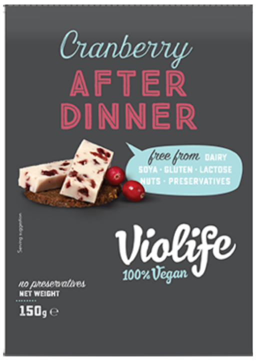

Ingredients:
Water, coconut oil (18%), modified starch, cranberry (9%), starch, sea salt, sunflower kernel grounded, flavourings, olive extract, colour: B-carotene, vitamin B12.

This vegan cheese contains protein 0.6g/100g.

If you had 70g of this how many exchanges would you count for this?

a. Not allowed
b. ½ exchange
c. 1 exchange
d. Exchange-free
e. Other amount (write here) __________________
f. Unsure

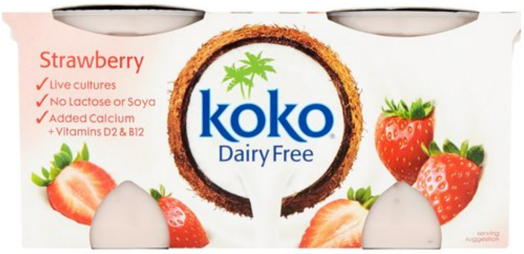

Ingredients:
Water, coconut milk (17%), strawberries (7%), sugar, thickeners (modified maize starch, pectin, guar gum), dextrose, glucose fructose syrup, concentrated strawberry juice, calcium phosphate, salt, Vitamin D2, natural flavouring, vitamin B12, colour (strawberry concentrate, carotene), non-dairy live cultures (S.Thermophulis, L.Bulgaricus).

This coconut yogurt contains protein 0.6g/100g and one tub is 125g.

How many exchanges would you count for 1 tub?

a. Not allowed
b. ½ exchange
c. 1 exchange
d. Exchange-free
e. Other amount (write here) __________________
f. Unsure

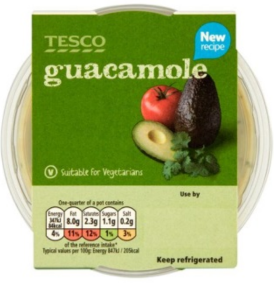

Ingredients:
Avocado, tomato, soured cream, onion, concentrated lime juice, coriander, antioxidant (ascorbic acid), garlic puree, red chilli, salt, rice starch.

This dip contains protein 1.4g/100g.

How many exchanges would be in 50g?

a. Not allowed
b. ½ exchange
c. 1 exchange
d. 1 ½ exchanges
e. Exchange-free
f. Other amount (write here) __________________
g. Unsure

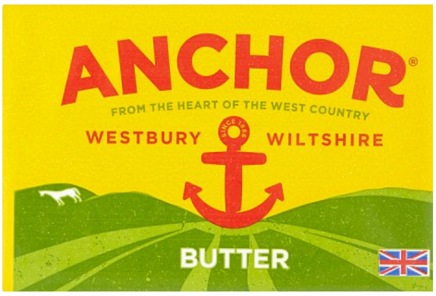

This butter contains protein 0.6g/100g.

Could you eat this?

a. Yes it's exchange-free
b. Not allowed
c. Could eat a small amount
d. I would count it as part of my exchanges
e. Other: (write here)___________________________
f. Unsure

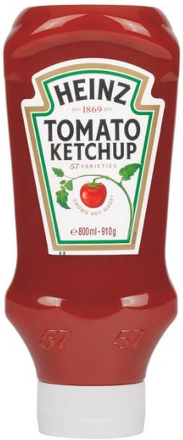

Ingredients: Tomatoes, spirit vinegar, sugar, salt, spice and herb extracts, spice.

This ketchup contains protein 1.2g/100g.

Could you eat this?

a. Yes it's exchange-free because all the ingredients are exchange-free
b. Not allowed
c. Could eat a small amount
d. I would count it as part of my exchanges
e. Other: (write here)___________________________
f. Unsure

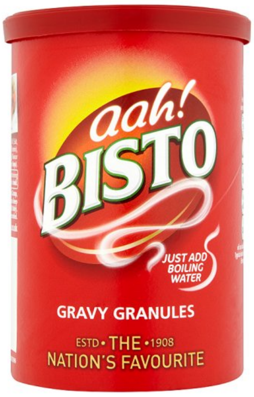

These gravy granules contains protein <0.5g/100 ml.

Could you eat this?

a. Yes it's exchange-free
b. Not allowed
c. Could eat a small amount
d. I would count it as part of my exchanges
e. Other: (write here)___________________________
f. Unsure

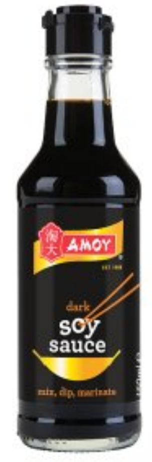

This soy sauce contains protein 1.3g/100g.

Could you eat this?

a. Yes it's exchange-free
b. Not allowed
c. Could eat a small amount
d. I would count it as part of my exchanges
e. Other: (write here)___________________________
f. Unsure

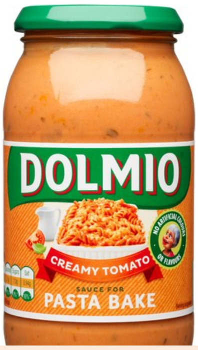

Ingredients:
Tomatoes (75%), onions, fresh cream (4%), sunflower oil, sugar, modified maize starch, white wine, tomato paste (1%), butterfat, cheddar cheese, salt, basil, white wine vinegar, lactose, acidity regulator (lactic acid), natural flavouring (celery), palm fat, herbs, spices, milk proteins, stabiliser (xanthan gum), antioxidant (rosemary extract).

This jar of sauce contains protein 1.1g/100g.

How many exchanges would be in 125g?

a. Not allowed
b. 1 exchange
c. 1½ exchanges
d. Exchange-free
e. Other amount (write here) ___________________
f. Unsure

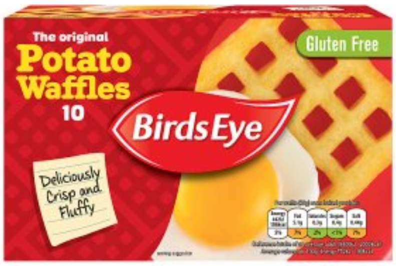

	Per 100g	Per waffle (58g)
Protein	2.5 g	1.4g

Ingredients:
Potato (87%), rapeseed oil, potato granules, potato starch, salt, stabiliser (E464), white pepper.

How many exchanges is 1 waffle (58g)?

a. Not allowed
b. 1 exchange
c. 1½ exchanges
d. 2 exchanges
e. 2.5 exchanges
f. Exchange-free
g. Other amount (write here) ______________
h. Unsure

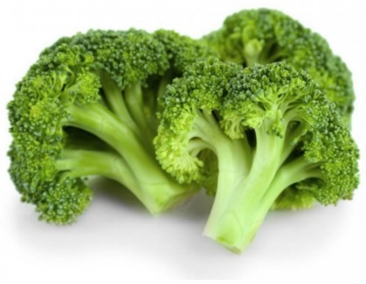

How much of this vegetable provides one exchange?

a. It's exchange-free
b. Not allowed
c. _________ grams

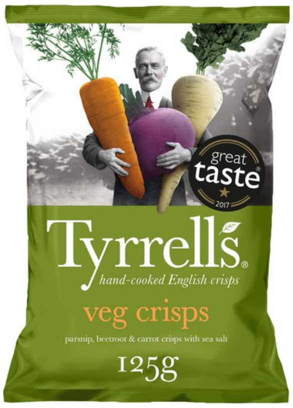

Ingredients:
Mixed root vegetables (parsnip, beetroot, carrot, 66% sunflower oil, sea salt.

These crisps contain protein 4.1g/100g.

If you ate 25g, how many exchanges would this be?

a. Not allowed
b. 1 exchange
c. 1½ exchanges
d. 4 exchanges
e. Exchange-free because all the ingredients are exchange-free.
f. Other amount (write here) ___________________
g. Unsure

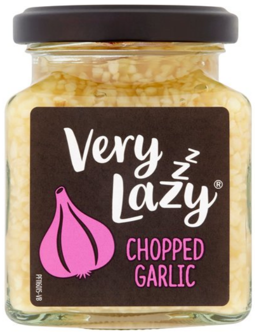

Ingredients:
White wine vinegar (sulphites), dried garlic.

This garlic provides protein 4.7g/100g.

Can you eat this?

a. Yes it's exchange-free because all the ingredients are exchange-free and I will only use a small amount
b. Not allowed
c. Could eat a small amount
d. I would count it as part of my exchanges
e. Other: (write here)___________________________
f. Unsure

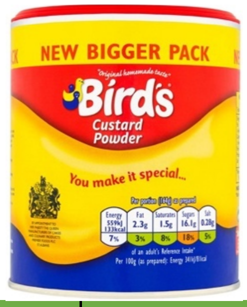

Ingredients:
Maize starch, salt, flavouring, colour.

	**Per 100g as prepared with semi-skim milk**	**Per portion (164g) as prepared with semi-skim milk**
Protein	3.0 g	4.9g

Can you have this custard powder?

a. Yes it's exchange-free if made up with low protein milk, because all the ingredients are exchange-free.
b. Not allowed
c. Could eat a small amount
d. I would count it as part of my exchanges
e. Other: (write here)___________________________
f. Unsure

What would you add to this product to make it up?

____
